# The role of intracellular calcium and Rho kinase pathways in G protein-coupled receptor-mediated contractions of urinary bladder urothelium and lamina propria

**DOI:** 10.1152/ajpcell.00441.2022

**Published:** 2023-01-23

**Authors:** Charlotte Phelps, Russ Chess-Williams, Christian Moro

**Affiliations:** Centre for Urology Research, Faculty of Health Sciences and Medicine, Bond University, Gold Coast, Queensland, Australia

**Keywords:** Gq receptors, mucosa, overactive bladder, rho kinase, underactive bladder

## Abstract

The influence of extracellular and intracellular calcium on smooth muscle contractile activity varies between organs. In response to G protein-coupled receptor (GPCR) stimulation, the urinary bladder detrusor muscle has shown a 70% dependence on extracellular calcium, whereas the urothelium and lamina propria (U&LP) has a 20%–50% dependence. However, as this only accounts for partial contractile activity, the contribution of intracellular calcium and calcium sensitization pathways remains unclear. This study assessed the role of intracellular signaling pathways on GPCR-mediated urinary bladder U&LP contraction. Porcine U&LP responses to activation of the G_q/11_-coupled muscarinic, histamine, 5-hydroxytryptamine (serotonin), neurokinin, prostaglandin, and angiotensin II receptors were assessed with three selective inhibitors of store-released intracellular calcium, 2-aminoethyl diphenylborinate (2-APB), cyclopiazonic acid (CPA), and ruthenium red, and three Rho kinase inhibitors, fasudil, Y-27632, and GSK269962. There was no discernible impact on receptor agonist-induced contractions of the U&LP after blocking intracellular calcium pathways, suggesting that this tissue is more sensitive to alterations in the availability of extracellular calcium. However, an alternative mechanism of action for GPCR-mediated contraction was identified to be the activation of Rho kinase, such as when Y-27632 significantly reduced the GPCR-mediated contractile activity of the U&LP by approximately 50% (*P* < 0.05, *n* = 8). This suggests that contractile responses of the bladder U&LP do not involve a significant release of calcium from intracellular stores, but that G_q/11_-coupled receptor activation causes calcium sensitization via Rho kinase. This study highlights a key role for Rho kinase in the urinary bladder, which may provide a novel target in the future pharmaceutical management of bladder contractile disorders.

## Graphical abstract

**Figure:**
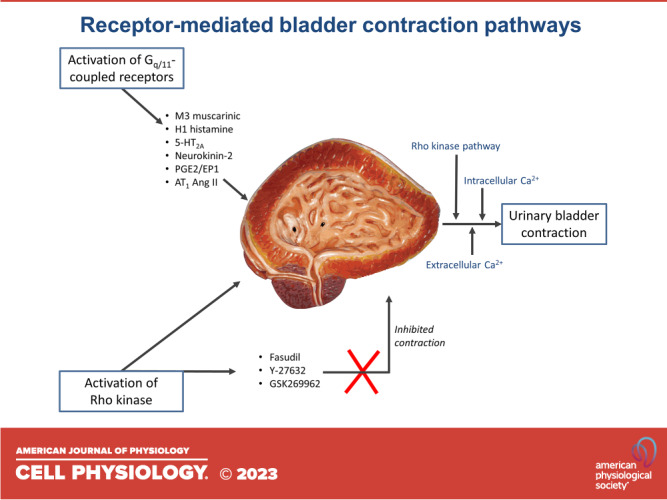

## INTRODUCTION

The most common front-line target in the pharmaceutical management of bladder contractile disorders is the G_q/11_-coupled M3 muscarinic receptor (1). However, in many cases, the prescription of muscarinic receptor antagonists for overactive bladder and muscarinic receptor agonists for underactive bladder, is ineffective, with most patients ceasing their pharmaceutical regimens (2) due to lower-than-expected treatment outcomes or recurring side effects (1, 3). This presents a need to further the current understanding of receptor systems involved in urinary bladder contractions. Investigating the influence of receptor-mediated contractions on bladder activity may also identify potential therapeutic targets and novel treatments for underactive and overactive bladder (1). There has been a particular focus on the detrusor smooth muscle in the pharmaceutical management of bladder contractile disorders. However, the luminal urothelial layer and underlying lamina propria, also termed the “bladder mucosa”, has the potential to influence whole bladder activity (4). It has been hypothesized that dysfunction of receptor systems within the urothelium and lamina propria (U&LP) may be an underlying cause in a variety of bladder disorders (5).

The contraction of the urinary bladder to stimulate emptying is primarily initiated by the release of acetylcholine from cholinergic nerves, which stimulates muscarinic receptors and triggers intracellular signaling cascades. In contrast to the human detrusor, where over 70% of G protein-coupled receptor (GPCR)-activated contraction of the smooth muscle is known to be dependent on extracellular sources of calcium (Ca^2+^) (6), extracellular Ca^2+^ only accounts for 20%–50% of GPCR-mediated contractions in the porcine U&LP (7). This lowered dependence on extracellular sources presents the potential for intracellular signaling pathways to play an important functional role within the U&LP. Activation of G_q/11_-coupled receptors activates phospholipase C and catalyzes the hydrolysis of phosphatidylinositol biphosphate to generate inositol trisphosphate (IP_3_) and diacylglycerol (DAG) (8). These second messengers propagate and amplify the G_q/11_-mediated signal with Ca^2+^ mobilization by Ca^2+^ release from intracellular stores and DAG-dependent protein kinase C activation (9). An increase in the intracellular Ca^2+^ concentration is typically the primary stimulus for smooth muscle contraction, where Ca^2+^ binds to calmodulin, and the resulting complex activates myosin light chain kinase (MLCK), which phosphorylates MLC and promotes the interaction of myosin with actin for subsequent contraction. An elevated concentration of intracellular Ca^2+^ is essential for the normal contractile activity of the urinary bladder. Levels of Ca^2+^ in the cytosol are dependent on the influx of extracellular Ca^2+^ via plasma membrane Ca^2+^ channels, as well as Ca^2+^ release from intracellular stores. However, there remains uncertainty about whether this intracellular Ca^2+^ concentration is due to Ca^2+^ influx entering the membrane from extracellular sources or the release of Ca^2+^ from the sarcoplasmic reticulum (SR) (6, 10, 11).

The Rho kinase pathway also plays a role in urinary bladder contraction by modifying the sensitivity of contractile and regulatory proteins to intracellular Ca^2+^ concentrations (12). Rho kinase is the major Ca^2+^-dependent pathway in contraction and has been shown to play an important role in the spontaneous contractile activity of the intact urinary bladder (13). The Ca^2+^ sensitization events inhibit myosin light chain phosphatase (MLCP) and cause a leftward shift of the Ca^2+^-force response curve at consistent Ca^2+^ and MLCK levels. Agonists acting on G_q/11_-coupled receptors induce Ca^2+^ sensitization via the activation of Rho kinase. This occurs when the small monomeric G protein, RhoA, activated from GTP binding, phosphorylates the regulatory subunit of MLCP, which inhibits phosphatase activity and leads to sustained contractions (14). In the human detrusor muscle, it has been observed that muscarinic receptor-mediated contraction involves increases in intracellular Ca^2+^ concentrations, and also increases the Ca^2+^ sensitivity of contractile apparatus in a Rho kinase- and protein kinase C-dependent manner (15).

To investigate the contribution of intracellular Ca^2+^-dependent and Ca^2+^-independent sources for receptor-mediated U&LP contractions, selective pharmacological inhibitors for these pathways were chosen. 2-Aminoethyl diphenylborinate (2-APB) is a membrane-penetrable inhibitor of IP_3_-induced Ca^2+^ release (16). Cyclopiazonic acid (CPA) is an inhibitor of the Ca^2+^-ATPase pump of the SR (17). Ruthenium red has a primary effect on the mitochondrial Ca^2+^ uniporter (18), and a secondary effect on impacting Ca^2+^ efflux from the SR via IP_3_-gated and ryanodine receptors (19). Fasudil (20), Y-27632 (21), and GSK269962 (*N*-[3-[[2-(4-amino-1,2,5-oxadiazol-3-yl)-1-ethyl-1*H*-imidazo[4,5-*c*]pyridin-6-yl]oxy]phenyl]-4-[2-(4-morpholinyl)ethoxy]benzamide) (22) are selective Rho-associated protein kinase inhibitors.

There is increasing evidence to support a prominent role of the U&LP in overall bladder contractile activity (23, 24). In particular, this tissue layer may release mediators such as acetylcholine, ATP (25), or prostaglandins (26) to stimulate spontaneous contractions, or impact the activity of the underlying detrusor smooth muscle (27). However, most past research into bladder contraction has focused on the activity of the detrusor (28), leaving a limited understanding of mechanisms taking place within the U&LP. The aim of this study is to identify intracellular pathways responsible for mediating contractions across a range of G_q/11_-coupled receptor systems in the bladder U&LP. This includes identifying whether intracellular mechanisms of action are different between muscarinic, histamine, 5-hydroxytryptamine (5-HT), neurokinin, prostaglandin, and angiotensin receptors. The influence that both Ca^2+^ release from intracellular sources, as well as Ca^2+^-independent pathways through the Rho kinase system, plays in urinary bladder U&LP contractions will be assessed.

## MATERIALS AND METHODS

### Tissue Collection and Preparation

Urinary bladders were obtained from Large White-Landrace-Duroc pigs (6 mo old, 80 kg live weight) from the local abattoir. As no animals were bred, harmed, culled, interfered, or interacted with as part of this research project, animal ethics approval was not required for this use of offal (29). After collection, tissues were transported in a portable cooler in cold Krebs–Henseleit bicarbonate solution (“Krebs,” composition: NaCl 118.4 mM, NaHCO_3_ 24.9 mM, d-glucose 11.7 mM, KCl 4.6 mM, MgSO_4_ 2.41 mM, CaCl_2_ 1.9 mM, and KH_2_PO_4_ 1.18 mM), maintained at 4°C, to the University research facilities and used within 5 h of the organ’s retrieval.

Adjacent paired strips (2 cm × 0.5 cm) of isolated U&LP were separated from the underlying detrusor smooth muscle of whole urinary bladders (30). Strips of U&LP were mounted and suspended in 10 mL organ baths (Labglass, Brisbane, Australia) containing Krebs solution maintained at 37°C and constantly perfused with carbogen gas (95% oxygen and 5% carbon dioxide). Each bath was washed with warmed fresh Krebs solution a total of three times before experimentation. The tension placed on the tissues was manually adjusted to 20 mN using a moveable transducer positioner with a fine adjustment level. The mean ± standard deviation weight of isolated U&LP tissues used in this study was 160 ± 20 mg (average taken from 578 tissue samples).

### Pharmaceutical Agents

Carbamylcholine chloride (carbachol), histamine dihydrochloride (histamine), 2-aminoethyl diphenylborinate (2-APB), and fasudil hydrochloride (fasudil) were obtained from Sigma Aldrich (St. Louis, MO). Neurokinin-A (NKA), cyclopiazonic acid (CPA), and GSK269962 (GSK; *N*-[3-[[2-(4-amino-1,2,5-oxadiazol-3-yl)-1-ethyl-1*H*-imidazo[4,5-*c*]pyridin-6-yl]oxy]phenyl]-4-[2-(4-morpholinyl)ethoxy]benzamide) were obtained from Tocris Bioscience (Bristol, UK). 5-Hydroxytryptamine (5-HT) was obtained from Toronto Research Chemicals (Toronto, ON, Canada), and Y-27632 hydrochloride (Y-27632) from AdooQ BioScience (Irvine, CA). Prostaglandin-E2 (PGE2), angiotensin-II (ATII), and ruthenium red were obtained from Cayman Chemicals (Ann Arbor, MI). All ingredients for the Krebs solution were from Sigma Aldrich (St. Louis, MO). PGE2 was dissolved in 100% ethanol, and CPA and fasudil were dissolved in 100% dimethyl sulfoxide (DMSO). All other pharmaceutical agents were soluble in distilled water. Concentrations selected for the agonists and antagonists were chosen based on their selectivity at each receptor and consistent with concentrations used in previous studies utilizing porcine tissue. In all cases, the receptor agonist concentration used induced submaximal contraction. For this assessment, preliminary studies using concentration-response curves were performed to identify maximal contractions. The agonist concentrations were then chosen as the dose that induced ∼80% of this contraction, allowing for a strong, submaximal contraction of the tissue.

### Measurements of Contractile Activity

A single dose of carbachol (1 µM, muscarinic receptor agonist), histamine (100 µM), 5-hydroxytryptamine (100 µM), neurokinin-A (300 nM), prostaglandin-E2 (10 µM), or angiotensin-II (100 nM) was applied to the U&LP tissue strips following a 30-min equilibration period. Baseline tension (millinewtons, mN), frequency (cycles per minute, cycles/min), and amplitude (mN) of spontaneous phasic contractions was measured before and after the application of the GPCR agonist with an isometric force transducer (MCT050/D, ADInstruments, Castle Hill, Australia) and recorded on a PowerLab system using LabChart v7 software (ADInstruments). Throughout this manuscript baseline change in tension (ΔmN) is related specifically to baseline force.

2-Aminoethyl diphenylborinate (300 µM), ruthenium red (10 µM), fasudil (30 µM), Y-27632 (1 µM), and GSK269962 (1 µM) were applied separately to tissues for a 30-min incubation period before a single dose of GPCR agonist was added. In separate experiments using the intracellular Ca^2+^-ATPase pump inhibitor, CPA (10 µM), a wash-out method was employed to exhaust the intracellular Ca^2+^ stores from the SR to avoid reuptake by Ca^2+^-ATPase. Initially, tissues were washed with warm Krebs containing a DMSO vehicle control (totaling 0.03% DMSO) for the control tissues, or CPA (10 µM) for experimental tissues and equilibrated for 30 min. After equilibration, a single dose of carbachol (1 µM) was added and when contraction reached its peak, tissues were washed with either control or CPA Krebs three times to remove any excess agonist. This process was repeated a total of three times for all tissues, and after the third wash, a single dose of a select GPCR agonist was added. During the wash-out procedure, spontaneous phasic activity was disrupted, and as such, only baseline tension (mN) was recorded and analyzed for CPA studies.

Data were graphed and analyzed using Prism 9.4.1 for Windows (GraphPad Software, La Jolla, CA). All values were reported as mean change ± standard deviation (SD). Statistical analysis was conducted using paired Student’s two-tailed *t* tests when comparing tissues with their paired, direct control tissues. On one occasion (see *Spontaneous Phasic Activity of the U&LP in Response to GPCR Agonists*), an unpaired Student’s *t* -test was applied when overall contractile responses between all receptor agonists were assessed. For all statistical analyses, *P* < 0.05 was considered statistically significant. Throughout this manuscript, *n* (number of tissues) values quoted are from paired tissue strips, and as such, the number of animals (*N*) used can be calculated using n÷2. This study followed an exploratory nature (31, 32) with *P* values presented used for descriptive analysis, rather than the formal testing of a prespecified statistical null hypothesis. Effect sizes (33) were calculated using an unbiased estimate of Cohen’s *d* (34) and values determined small (*d* = 0.2), medium (*d* = 0.5), and large (*d* = 0.8).

## RESULTS

### Spontaneous Phasic Activity of the U&LP in Response to GPCR Agonists

Spontaneous phasic activity was observed in strips of U&LP in the absence of any stimulation at a means ± SD baseline tension of 19.32 ± 4.09 mN (*n* = 241), frequency of 4.03 ± 0.91 cycles/min (*n* = 241), and amplitude of 6.29 ± 0.24 mN (*n* = 241). In all cases, responses to GPCR agonists increased baseline tension (*P* < 0.001) and the frequency of spontaneous phasic contractions (*P* < 0.05). A decrease in the amplitude of contractions was observed with all agonists except histamine and NKA (Table 1).

**Table 1.** Summary of the changes in U&LP tension, frequency, and amplitude of contraction after the addition of a single dose of GPCR agonist

Agonist	Concentration	Δ Tension, mN	Δ Frequency, cycles/min	Δ Amplitude, mN	*n*
Carbachol	1 µM	35.39 ± 15.43***	0.90 ± 1.38***	−2.75 ± 4.30***	38
Histamine	100 µM	12.63 ± 7.67***	0.40 ± 0.87**	−0.18 ± 2.74	42
5-HT	100 µM	53.33 ± 16.22***	2.33 ± 3.27***	−4.36 ± 2.64***	39
NKA	300 nM	21.18 ± 10.97***	0.33 ± 0.98*	−0.99 ± 3.40	40
PGE2	10 µM	18.88 ± 5.93***	0.97 ± 1.41***	−1.29 ± 2.23***	42
ATII	100 nM	12.04 ± 4.62***	0.73 ± 1.19***	−0.88 ± 1.25***	40

Data are represented as means ± SD. ATII, angiotensin-II; GPCR, G protein-coupled receptor; 5-HT, 5-hydroxytryptamine; NKA, neurokinin-A; PGE2, prostaglandin-E2; U&LP, urothelium and lamina propria.

*P < 0.05, **P < 0.01, ***P < 0.001 (unpaired Student’s two-tailed *t* test).

### Influence of Agents That Reduce Intracellular Ca^2+^ Responses on Spontaneous Phasic Contractions

#### Influence of 2-APB on baseline tension, frequency, and amplitude.

The responses to six GPCR agonists in the presence of an inhibitor of IP3-induced Ca^2+^ release, 2-APB, were observed. In the presence of 2-APB, increases in the baseline tension of spontaneous phasic contractions in response to histamine (100 µM) was significantly inhibited by 62% (from 16.35 ± 10.21 mN to 6.18 ± 4.52 mN, *n* = 12, *P* < 0.001). There was no difference to the change in baseline tension for responses to carbachol, 5-HT, NKA, PGE2, or ATII in the presence of 2-APB (Table 2). PGE2 (10 µM) in the presence of 2-APB caused a greater increase in the frequency of spontaneous phasic contractions (*n* = 8, *P* < 0.01) and a decrease in the amplitude of contraction peaks (*n* = 8, *P* < 0.01) compared with controls. In addition, ATII (100 nM) in the presence of 2-APB, was observed to decrease the frequency of spontaneous phasic contractions (*n* = 8, *P* < 0.01) with no change to amplitude.

**Table 2.** The effect of 2-APB (300 µM) on U&LP tension, frequency, and amplitude of contraction

Agonist (*n*)	Conc.	Control	+ 2-APB	*P* Value
		Δ Tension, mN		
Carbachol (8)	1 µM	28.99 ± 9.89	32.62 ± 9.88	0.27
**Histamine** (12)	**100 µM**	**16.35 ± 10.21**	**6.18 ± 4.52**	0.001***
5-HT (8)	100 µM	53.43 ± 18.16	48.42 ± 20.68	0.47
NKA (8)	300 nM	16.88 ± 12.43	14.64 ± 9.08	0.70
PGE2 (8)	10 µM	21.15 ± 4.39	27.99 ± 10.84	0.13
ATII (8)	100 nM	8.59 ± 2.96	10.19 ± 4.63	0.43
		Δ Frequency, cycles/min		
Carbachol (8)	1 µM	0.58 ± 1.18	1.09 ± 1.69	0.22
Histamine (12)	100 µM	0.14 ± 0.90	1.40 ± 2.80	0.12
5-HT (8)	100 µM	0.78 ± 2.26	2.15 ± 1.81	0.23
NKA (8)	300 nM	−0.49 ± 0.98	−0.84 ± 3.78	0.78
**PGE2** (8)	**10 µM**	**0.30 ± 1.11**	**1.45 ± 0.89**	0.006**
**ATII** (8)	**100 nM**	**0.72 ± 0.96**	**−0.58 ± 0.97**	0.004**
		Δ Amplitude, mN		
Carbachol (8)	1 µM	−1.04 ± 2.62	−1.86 ± 1.54	0.42
Histamine (12)	100 µM	−1.28 ± 2.18	−1.00 ± 1.56	0.71
5-HT (8)	100 µM	−3.41 ± 2.22	−4.76 ± 2.38	0.28
NKA (8)	300 nM	−0.60 ± 2.07	−1.01 ± 1.06	0.53
**PGE2** (8)	**10 µM**	**−1.19 ± 0.88**	**−2.95 ± 1.02**	0.006**
ATII (8)	100 nM	−0.36 ± 0.89	0.82 ± 1.17	0.08

Data are represented as means ± SD. 2-APB, 2-aminoethyl diphenylborinate; ATII, angiotensin-II; 5-HT, 5-hydroxytryptamine; NKA, neurokinin-A; PGE2, prostaglandin-E2; U&LP, urothelium and lamina propria.

**P < 0.01, ***P < 0.001 (paired Student’s two-tailed *t* test). Bold font designates significant difference.

#### Influence of CPA on spontaneous phasic contractions in U&LP.

After equilibration with CPA (10 µM), a wash-out method was undertaken to deplete SR intracellular Ca^2+^ stores from tissues. Baseline tension responses to receptor activation were observed to decrease after each tissue wash-out, both in the absence (control) and presence of CPA, indicating an effective depletion of Ca^2+^ stores. Following the wash-out method, in the presence of CPA (10 µM, *n* = 8 for all), there was no difference between responses to 1 µM carbachol, 100 µM histamine, 100 µM 5-HT, 10 µM PGE2, or 100 nM ATII compared with control contractions (all contractions *P* < 0.001 in both the presence and absence of CPA). However, tension responses to 300 nM NKA were enhanced in the presence of CPA (NKA: Δ26.11 ± 7.93 mN; NKA + CPA: Δ47.80 ± 13.00 mN; *P* < 0.001, *n* = 8).

#### Influence of ruthenium red on spontaneous phasic contractions in U&LP.

In the presence of the nonspecific inhibitor of intracellular Ca^2+^ signaling, ruthenium red (10 µM), the baseline tension responses to histamine (100 µM) were inhibited by 61% (histamine alone: Δ 5.34 ± 2.52 mN; histamine + ruthenium red: Δ 2.08 ± 4.32 mN; *P* < 0.05, *n* = 8). However, there were no significant differences between the absence and presence of ruthenium red for frequency and amplitude of spontaneous phasic contractions in response to histamine. In all other cases, there were no differences between observed responses in the absence and presence of ruthenium red (10 µM, *n* = 8 for all) to carbachol (1 µM), 5-HT (100 µM), NKA (300 nM), PGE2 (10 µM), or ATII (100 nM).

### Influence of Rho Kinase Inhibitors on Spontaneous Phasic Contractions

#### Influence of fasudil on spontaneous phasic contractions in U&LP.

In the presence of the Rho kinase inhibitor, fasudil (30 µM), the baseline tension for all tissues decreased during the 30-min equilibration period by ∼8.63 mN (*n* = 50, *P* < 0.001). No change was observed from the addition of a DMSO vehicle control during equilibration.

##### Baseline tension responses to agonists.

**Figure 1.**
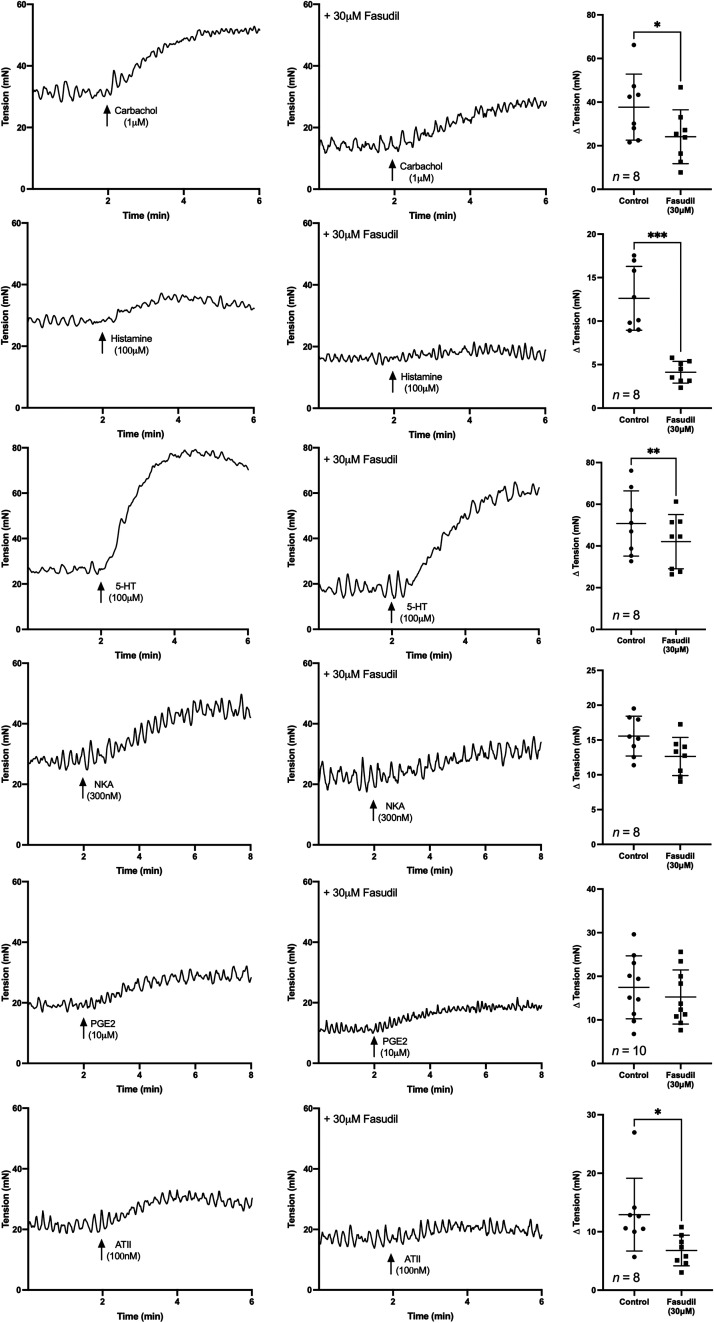
The effect of fasudil (30 µM) on urothelium and lamina propria (U&LP) baseline tension and responses to G protein-coupled receptor (GPCR) agonists (means ± SD). *P < 0.05, **P < 0.01, ***P < 0.001 (paired Student’s two-tailed *t* test). ATII, angiotensin-II; 5-HT, 5-hydroxytryptamine; NKA, neurokinin-A; PGE2, prostaglandin-E2.

After activation of the six GPCRs with agonists, the baseline tension of the U&LP increased (Fig. 1). In the presence of the Rho kinase inhibitor fasudil (30 µM), increases in the baseline tension of contractions in response to receptor agonists was inhibited for all except NKA (300 nM) and PGE2 (10 µM). Upon observation, the effect on the responses to histamine (100 µM, *n* = 8) appeared significantly greater than its effect on 5-HT (100 µM, *n* = 8), where fasudil inhibited contraction by 67% for histamine (histamine alone: Δ 12.61 ± 3.67 mN; histamine + fasudil: Δ 4.12 ± 1.25 mN, *P* < 0.001) and 17% for 5-HT (5-HT alone: Δ 50.79 ± 15.65 mN; 5-HT + fasudil: Δ 42.06 ± 13.00 mN, *P* < 0.01).

##### Frequency and amplitude of phasic contractions.

The frequency and amplitude of spontaneous phasic contractions produced by the U&LP tissues in response to each of the receptor agonists were investigated in the absence and presence of 30 µM fasudil (Table 3). No change to frequency was observed in response to carbachol, 5-HT, NKA, PGE2, or ATII in the presence of fasudil. In addition, fasudil did not have any effect on the amplitude of contractile activity in response to histamine, NKA, PGE2, or ATII. However, fasudil (30 µM) reduced the frequency of spontaneous phasic contractions in response to histamine (100 µM, *n* = 8, *P* < 0.01) compared with the control tissues. Furthermore, in response to carbachol (1 µM, *n* = 8, *P* < 0.01) and 5-HT (100 µM, *n* = 8, *P* < 0.05), the amplitude of spontaneous phasic contractions was altered in the presence of fasudil compared with in the absence of the Rho kinase inhibitor.

**Table 3.** The effect of fasudil (30 µM) on U&LP tension, frequency, and amplitude of contraction

Agonist (*n*)	Conc.	Control	+ Fasudil	*P* Value
		Δ Frequency, cycles/min		
Carbachol (8)	1 µM	1.51 ± 2.15	0.46 ± 1.29	0.17
**Histamine** (8)	**100 µM**	**0.64 ± 0.66**	**0.06 ± 0.46**	0.009**
5-HT (8)	100 µM	2.30 ± 1.60	2.27 ± 2.98	0.97
NKA (8)	300 nM	0.52 ± 0.88	0.29 ± 1.49	0.75
PGE2 (10)	10 µM	0.59 ± 1.43	**−**0.01 ± 1.75	0.38
ATII (8)	100 nM	**−**0.11 ± 0.61	0.26 ± 0.66	0.32
		Δ Amplitude, mN		
**Carbachol** (8)	**1 µM**	**−4.40 ± 3.52**	**1.24 ± 1.55**	0.006**
Histamine (8)	100 µM	1.39 ± 3.24	0.16 ± 1.64	0.46
**5-HT** (8)	**100 µM**	**−3.74 ± 2.11**	**−0.44 ± 3.78**	0.03*
NKA (8)	300 nM	**−**0.86 ± 1.43	**−**0.67 ± 3.28	0.87
PGE2 (10)	10 µM	**−**0.05 ± 1.81	**−**0.13 ± 2.23	0.88
ATII (8)	100 nM	**−**0.94 ± 0.66	**−**0.49 ± 1.40	0.42

Data are represented as means ± SD. ATII, angiotensin-II; 5-HT, 5-hydroxytryptamine; NKA, neurokinin-A; PGE2, prostaglandin-E2; U&LP, urothelium and lamina propria.

*P < 0.05, **P < 0.01 (paired Student’s two-tailed *t* test). Bold font designates significant difference.

#### Influence of Rho kinase inhibitor Y-27632 on spontaneous phasic contractions in U&LP.

##### Baseline tension responses to agonists.

**Figure 2.**
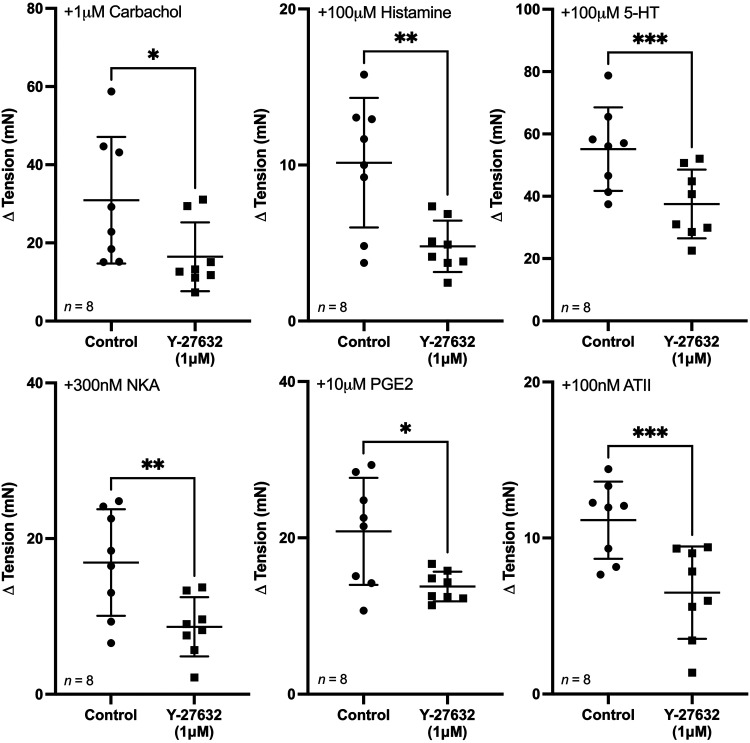
Urothelium and lamina propria (U&LP) baseline tension responses (means ± SD) to receptor agonists in the absence (control) and presence of the Rho kinase inhibitor Y-27632 (1 µM). *P < 0.05, **P < 0.01, ***P < 0.001 (paired Student’s two-tailed *t* test). ATII, angiotensin-II; 5-HT, 5-hydroxytryptamine; NKA, neurokinin-A; PGE2, prostaglandin-E2.

To further explore the involvement of Rho kinase in mediating GPCR-induced contractions, the Rho kinase inhibitor Y-27632 was applied to the tissue before activation by any agonist. Y-27632 (1 µM) significantly inhibited the tonic contractions of the U&LP observed in response to tissue stimulation by all GPCR agonists. This inhibition to the baseline tension of contractions was around 50% (Fig. 2).

##### Frequency and amplitude of phasic contractions.

The frequency of responses to the GPCR agonists (*n* = 8 for all) were not altered in the presence of Y-27632 (1 µM). In addition, there was no change in the amplitude of spontaneous phasic contractions with Rho kinase inhibition after the addition of 1 µM carbachol, 100 µM histamine, 100 µM 5-HT, 300 nM NKA, and 100 nM ATII (*n* = 8 for all). However, the amplitude of spontaneous phasic activity was significantly different after stimulation with PGE2 (control: Δ −3.47 ± 3.11 mN; with Y-27632: Δ −0.51 ± 1.69 mN; *P* < 0.01, *n* = 8).

#### Influence of GSK269962 on spontaneous phasic contractions in U&LP.

**Figure 3.**
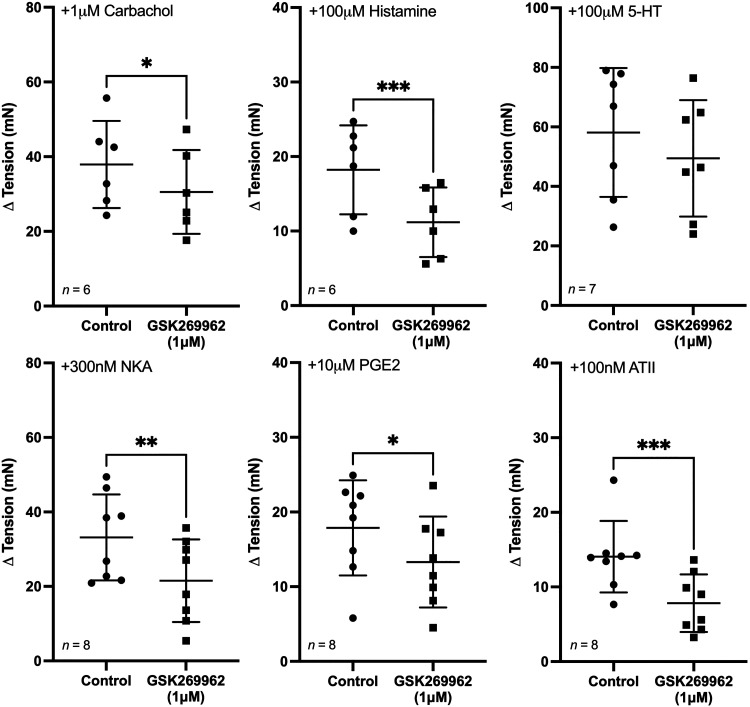
Urothelium and lamina propria (U&LP) baseline tension responses (means ± SD) to receptor agonists in the absence (control) and presence of GSK269962 (1 µM). *P < 0.05, **P < 0.01, ***P < 0.001 (paired Student’s two-tailed *t* test). ATII, angiotensin-II; 5-HT, 5-hydroxytryptamine; NKA, neurokinin-A; PGE2, prostaglandin-E2.

The Rho kinase inhibitor GSK269962 (1 µM) inhibited baseline tension responses to all GPCR agonists except for 5-HT (Fig. 3). The effect of GSK269962 on responses to carbachol, histamine, NKA, PGE2, and ATII appeared similar, inhibiting contractions by around 20%–40%. GSK269962 (1 µM) had no effect on either the frequency or amplitude of spontaneous contractions in response to any of the agonists.

## DISCUSSION

In smooth muscle contraction, both extracellular Ca^2+^ influx across the plasma membrane and intracellular Ca^2+^ release from the sarcoplasmic reticulum contribute to the increase in cytosolic Ca^2+^ concentration (35). In this current study, we support prior findings that the majority of contraction in the bladder U&LP is facilitated by the influx of extracellular Ca^2+^ (7). However, we also identified the GPCR-mediated activation of the Rho kinase pathway to be a major contributor to U&LP contractility.

The G_q/11_-coupled receptor subfamily has become a prominent area of research in urology. In particular, the urothelial and lamina propria M3 muscarinic (36), H1 histamine, 5-HT_2A_ (37), neurokinin-2 (38), EP1 prostaglandin E2 (39), and AT_1_ angiotensin II (7) receptors. In each case, activation of these receptors stimulated U&LP tissue contractions. Although evidence exists that there is an additional coupling of some of these receptor systems to alternative pathways, such as G_i_ across a variety of species (40), there remains the potential for a prominent contribution of G_q/11_-coupled receptors in a range of bladder contractile diseases, such as idiopathic overactive bladder or underactive bladder.

The influence of Ca^2+^ and intracellular signaling pathways for receptor-mediated contractions has been widely reported in the detrusor smooth muscle of the urinary bladder. The release of Ca^2+^ from the SR is an important step in the activation of the detrusor muscle, as studies using blockers of SR function have demonstrated that nerve- (41), agonist- (11), and stretch-induced contractions (42) of the urinary bladder are dependent on Ca^2+^ release from intracellular stores. Refilling of these intracellular Ca^2+^ stores in the porcine lower urinary tract occurs predominantly via the influx of Ca^2+^ through L-type Ca^2+^ channels (7, 43). However, other pathways may be involved, such as where depletion of intracellular Ca^2+^ stores due to GPCR activation has been shown to activate the transient receptor potential canonical protein family (44). The highly innervated lamina propria, which forms the distinct connective tissue layer between the urothelium and detrusor smooth muscle, accommodates a number of cells that could contribute to contraction, including myofibroblasts (45), interstitial-like cells (46), pericytes (47, 48), and muscularis mucosae (49, 50). It is cells within this layer that are thought to be responsible for inducing the spontaneous phasic activity and generating spontaneous Ca^2+^ transients.

M3 muscarinic receptor stimulation of the urinary bladder detrusor muscle can increase the sensitivity to Ca^2+^, leading to an influx of Ca^2+^ through L-type Ca^2+^ channels (51). However, the contribution of extracellular Ca^2+^ influx and intracellular store release to muscarinic receptor stimulation often differs between studies (10, 11) and species (6). Furthermore, other receptors systems, such as histamine (52), neurokinin (53), and purinergic receptors (54), have also been identified to depend on Ca^2+^ influences for urinary bladder contraction, however, there are inconsistencies in the reported contribution of extracellular and intracellular Ca^2+^ for these contractions. All previously reported literature has had a focus on the Ca^2+^ pathways controlling receptor-mediated contractions of the isolated detrusor smooth muscle layer or whole bladder preparations. Thus, this research is novel in reporting the influence of Ca^2+^ across a range of G_q/11_-coupled receptors in the isolated porcine U&LP.

In a previous study (7), we reported a strong dependence on extracellular Ca^2+^ for mediating muscarinic, histamine, 5-HT, neurokinin, prostaglandin, and angiotensin II contractions of the U&LP. This was shown to be consistent across two methods including inhibiting L-type Ca^2+^ channels with nifedipine as well as blocking Ca^2+^ entry into the U&LP with a nominally zero Ca^2+^ solution. The H1 histamine receptor, responsible for contraction in the porcine U&LP (55), may be of particular interest in future studies. Although most receptors identified in the current study had extracellular Ca^2+^ as the prominent source, histamine was unique, where baseline tension responses were inhibited by 60% from both 2-APB and ruthenium red. This suggests that the contraction to histamine receptor stimulation may be predominantly due to the influx of intracellular Ca^2+^, potentially through IP3-induced Ca^2+^ release. This directly correlates with the finding in our past study that identified inhibition of 40%–45% of the histamine response when extracellular sources of Ca^2+^ were removed (7).

CPA demonstrated minimal influence on the receptor-mediated activity of the U&LP. However, an interesting observation was the significant increase in baseline tension after the application of CPA in response to neurokinin-A receptor activation. A similar result was reported by Heppner et al. (50), where SR store depletion by CPA increased baseline tension of the bladder U&LP in guinea pigs, reflecting an elevated intracellular Ca^2+^ level caused by blockade of the SR Ca^2+^-ATPase pump. Although in our study we attempted to deplete intracellular Ca^2+^ levels with a wash-out method, neurokinin-induced contractile activity of the U&LP has demonstrated a strong dependence on both extracellular Ca^2+^, and the Rho kinase pathway, for contraction.

There has been a longstanding interest in investigating the role of Rho kinase, a Ca^2+^-independent mechanism, in receptor-mediated contractions (20, 56), with this pathway hypothesized to present a potentially novel target in the future treatments of bladder contractile disorders (57). In previous experiments, inhibition of extracellular Ca^2+^ influx reduced contractions, but not to a level where it was sufficient to entirely abolish the G_q/11_-mediated U&LP responses (7). However, with our current study contributing to this data, finding that the release of Ca^2+^ from intracellular stores held a minimal influence on agonist responses, the role of a Ca^2+^-independent pathway became of particular interest. It has been suggested that the U&LP may be involved in controlling carbachol-induced contractions of the bladder via the Rho kinase pathway, as fasudil demonstrates stronger inhibitory effects to contraction in urothelium-intact porcine tissue compared with denuded (20). In our study, we identified a prominent role for Rho kinase in mediating contractions in response to muscarinic, histamine, 5-HT, NKA, PGE2, and ATII receptor activation in the U&LP, which was demonstrated by inhibition of contractile responses in the presence of fasudil, Y-27632, and GSK. In particular, the influence of Rho kinase for receptor-mediated contractions was between 20% and 47% for carbachol, 39% and 67% for histamine, 17% and 32% for 5-HT, 35% and 49% for NKA, 25% and 34% for PGE2, and 42% and 47% for ATII. We suggest that this strong dependence on Rho kinase for G_q/11_-coupled receptor contractions may indicate G_q/11_ mediates RhoA activation in response to M3, H1, 5-HT_2A_, NK2, EP1, and AT_1_ stimulation in the U&LP. This is supported by the finding by Nakanishi et al. (58) that the U&LP expresses higher levels of RhoA mRNA and RhoA enzyme than the detrusor in the urinary bladder. One hypothesis for this link may be the G_q/11_-activated RhoGEF, p63RhoGEF, mediating RhoA activation (59). The unique activity observed in the U&LP, compared with many other smooth muscles, may be due to differences in the mechanisms facilitating its contraction. However, it is uncertain whether the contractions are mediated by myofibroblasts (45), interstitial-like cells (46), pericytes (47, 48), muscularis mucosae (49, 50), or a combination of these identified cells within the U&LP.

Across the three Rho kinase inhibitors, Y-27632 was the most effective and consistent inhibitor of the Ca^2+^ sensitization pathway, inhibiting responses to GPCR activation by 32%–53%. Fasudil also influenced all contractions (17%–67%) except those to NKA or PGE2. This is consistent with past findings identifying Y-27632 as the inhibitor with the higher affinity to Rho kinase (60) and selectivity across both ROCK-1 and ROCK-2 compared with fasudil. Alternatively, fasudil is less selective and comes with a risk of alternative actions on other kinases (61) as well as Ca^2+^ channels (62). More recently, a newer inhibitor has been developed with an even higher selectivity for ROCK, GSK269962 (63), and this was found to influence all contractions (20%–44%) except those in response to 5-HT. Of particular interest to future studies may be the finding that fasudil, yet not Y-27632 or GSK269962, was effective at inhibiting increases to the frequency of spontaneous contractions in the presence of histamine, and altering the amplitude of spontaneous activity in response to carbachol and 5-HT. It is unclear which mechanisms may be involved in this, but one hypothesis may be a variation in the effects of phosphatase on myosin light chain phosphorylation. With detrusor overactivity due to abnormal spontaneous contractions of the whole urinary bladder during filling, it may be of interest to further identify the range of potential mechanisms underlying this response.

The role of Ca^2+^ and Rho kinase in the mediation of G protein-coupled receptor contractions presents the potential for new and novel targets to be investigated for the pharmacological management of bladder contractile dysfunctions, such as underactive bladder. In elderly people with lower urinary tract symptoms, over 45% exhibit an underactive bladder, presenting this as an increasingly important and clinically relevant syndrome (64). However, there are currently no outcome-validated effective therapeutics for the management, treatment, or prevention of underactive bladder (1). In addition, underactive bladder can often coexist with overactive bladder, a disorder that has the most impact on patient quality of life among the lower urinary tract symptoms (65). However, until recently, there has been little research into the coexistent nature of overactive and underactive bladder (66), and without an established diagnostic criterion, or well-understood urodynamic correlates for this syndrome, it presents a prominent issue for patients suffering from bladder contractile disorders.

### Limitations and Future Direction

It should be noted that although three independent drugs were used to block the SR release of Ca^2+^ into the cytoplasm, there was a lack of inhibition on intracellular Ca^2+^ sources. The inhibitor 2-APB has been considered a reliable blocker of store-operated Ca^2+^ entry, however, may be an inconsistent inhibitor of IP3-induced Ca^2+^ release (67). To accommodate this concern, instead of drawing conclusions solely from 2-APB, we also investigated the influence of CPA and ruthenium red. One additional treatment that could have also been considered is xestospongin C (68), although due to a high procurement cost for the concentrations required, was not used in this study. It would also be of benefit to investigate the uniqueness of the U&LP responses compared with other smooth muscles, as well as identify which mechanisms might be responsible for the different influence of fasudil compared with Y-27632 or GSK269962 to histamine, carbachol, and 5-HT. There was some observed influence on contractile velocity in the study, for example, with 5-HT appearing to peak slower than other agonists, which would also be of interest for future studies to investigate.

### Conclusions

Ca^2+^-mediated contraction of the U&LP is typically stimulated by both extracellular Ca^2+^ influx and release from intracellular stores. However, in the urinary bladder U&LP, responses to G protein-coupled receptor stimulation are more sensitive to extracellular Ca^2+^ for the mediation of contractions, supported by the lack of any discernible impact on receptor agonist-induced contractions of the U&LP to 2-APB, CPA, and ruthenium red. An alternative mechanism of action for GPCR-mediated contraction for the muscarinic, histamine, 5-HT, neurokinin, prostaglandin, and angiotensin receptors was identified to be the activation of Rho kinase. The unique activity of the receptors investigated in this study presents novel directions for future research into the mechanisms underlying bladder contraction, and how these systems may be altered in disease states. The results suggest that Ca^2+^ regulation is important for activating the contractile machinery of the urinary bladder and highlight an equally important and complementary role for Rho kinase pathways. This presents potential novel targets for the pharmaceutical management of bladder disorders, in particular, overactive and underactive bladder.

## DATA AVAILABILITY

Data will be made available upon reasonable request.

## GRANTS

C. Phelps was supported by an Australian Government Research Training Program Scholarship.

## DISCLOSURES

No conflicts of interest, financial or otherwise, are declared by the authors.

## AUTHOR CONTRIBUTIONS

C.P. and C.M. conceived and designed research; C.P. performed experiments; C.P. and C.M. analyzed data; C.P., R.C.-W., and C.M. interpreted results of experiments; C.P. prepared figures; C.P. and C.M. drafted manuscript; C.P., R.C.-W., and C.M. edited and revised manuscript; C.P., R.C.-W., and C.M. approved final version of manuscript.
